# A safe procedure? The unusual case of a fatal airway obstruction by silicone during the production process of a tracheostomal epithesis in a 13-year-old boy

**DOI:** 10.1007/s00414-021-02720-x

**Published:** 2021-10-07

**Authors:** Daniel Wittschieber, Ronald Schulz, Peter F. Schmidt

**Affiliations:** 1 Institute of Legal Medicine, Jena University Hospital, Friedrich Schiller University Jena, Am Klinikum 1, 07743 Jena, Germany; 2 Institute of Legal Medicine, University Hospital Münster, Röntgenstraße 23, 48149 Münster, Germany

**Keywords:** Tracheostoma, Epithesis, Aspiration, Asphyxia, Malpractice

## Abstract

A tracheostomal epithesis is a plastic prosthesis that serves for sealing a tracheostoma and ensuring the position of the tracheostomy tube. The production of a tracheostomal epithesis requires an impression of the tracheostoma. To this end, silicone impression material is applied by an anaplastologist in and around the tracheostomal region, including the trachea. The blocked cuff of the tracheostomy tube serves to prevent aspiration of the material. We report on a 13-year-old boy who died during this procedure because the lower airways were obstructed with cured silicone. Forensic autopsy confirmed asphyctic suffocation as cause of death. Forensic physical investigation of the tracheostomy tube and its cuff revealed no structural or functional defects. Yet, the investigation results prove that the viscous silicone must have passed the cuff. To conclude, this case report demonstrates that the production of an impression of a tracheostoma is a procedure with a potentially lethal outcome. Hence, professional guidelines, including clear safety precautions, are urgently needed.

## Introduction

**Fig. 1:**
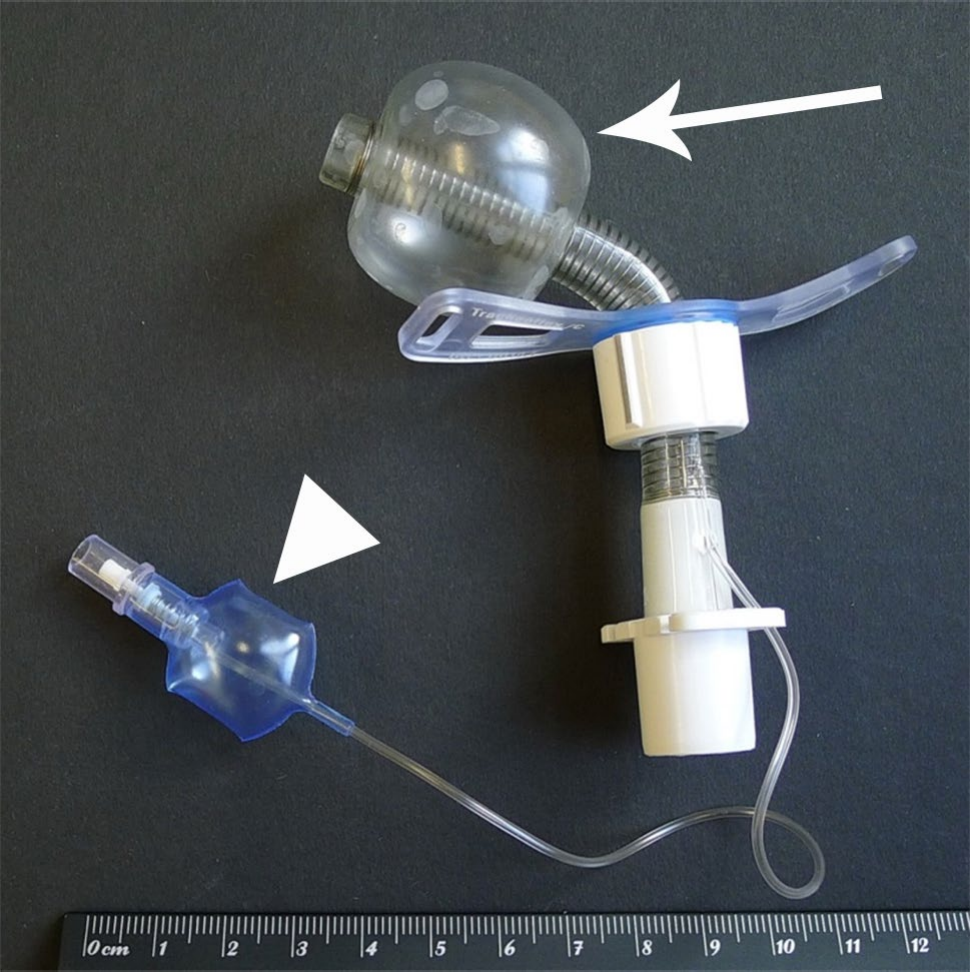
Unused tracheostomy tube with a maximally blocked cuff (arrow) indicated by the small blue control balloon (arrow head) which is maximally filled with air as well. Same model as used during the incident of the present case report

Tracheostomy represents one of the oldest and most frequently performed surgical procedures in critically ill adults and children [1]. Thereby, an artificial mouth-like opening of the anterior tracheal wall is created to establish an airway through the neck [1]. Then, this so-called *tracheostoma* (στόμα [ancient Greek] = mouth), which, strictly speaking, implies a permanent opening at the anterior neck area created by suturing skin flaps onto the tracheal walls [2], is usually maintained by inserting a so-called *tracheostomy tube* (Fig. 1) through the opening [1, 3]. Prolonged endotracheal intubation represents a typical indication for this procedure [3, 4].

In cases of long-term therapy, the anatomic conditions of the tracheostoma may become irregular or oversized in the course of time, for instance, due to chronic inflammation. If surgical modification failed or should be avoided, the irregularly formed tracheostoma can be corrected by insertion of an individually adjusted tracheostomal epithesis to seal the gap between the tracheostomy tube and tracheostoma.

Epitheses are individually fabricated prostheses made of glass, rubber, metal, or synthetic materials, and mostly serve as esthetical compensation of body defects, especially after maxillofacial surgery. In the case of the tracheostomal epithesis, this device has functional significance as well: sealing the tracheostoma and ensuring the position of the tracheostomy tube.

**Fig. 2:**
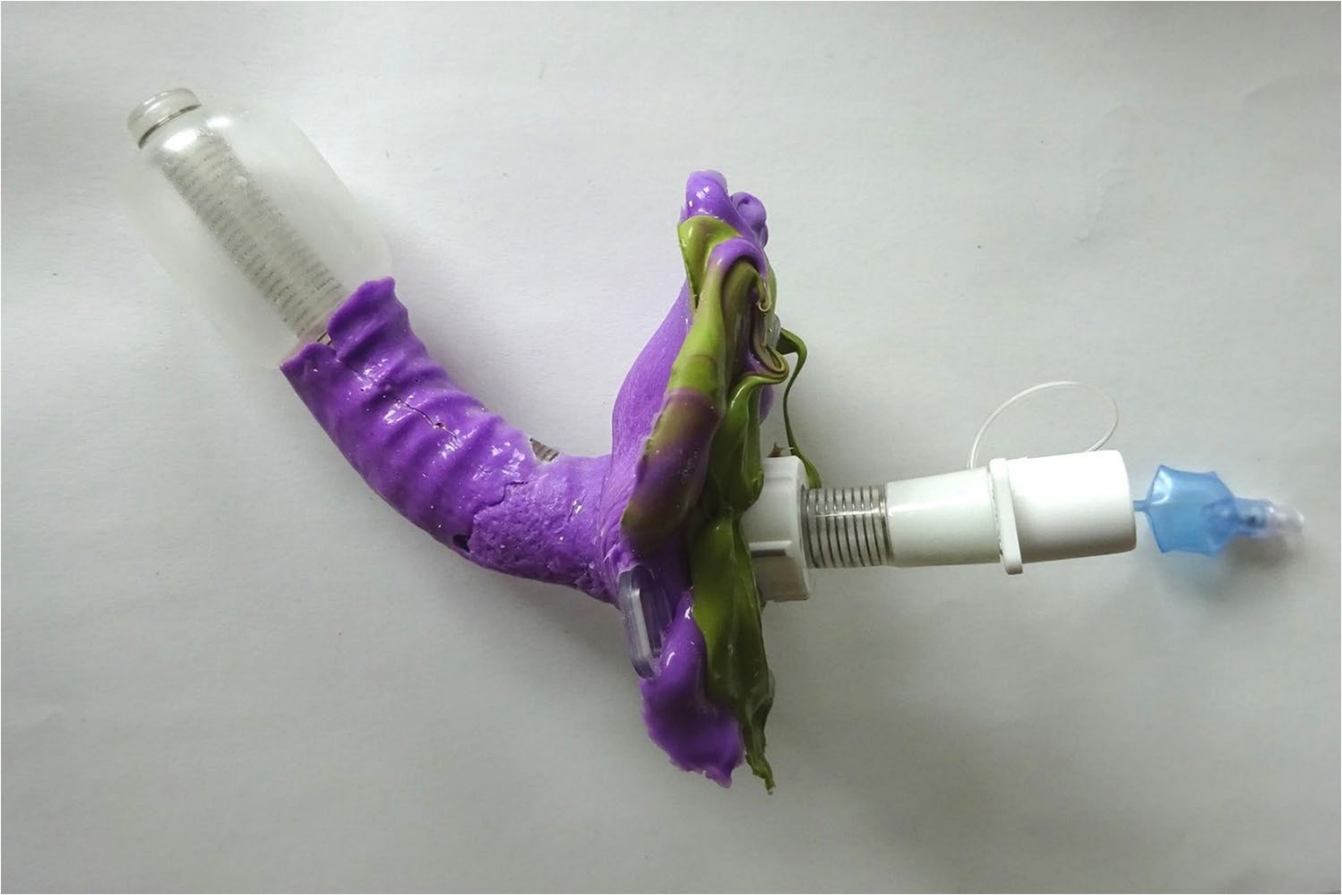
Used tracheostomy tube with cured purple and green silicone impression material, representing a negative impression of the tracheostoma and a small part of the trachea. The silicone cylinder ends at the upper end of the cuff. Figure shows the optimal end product in another case

The production of a tracheostomal epithesis requires an impression of the tracheostoma. This initial production step, which usually takes about 10 min, has been described and illustrated in detail by Bozzato et al. [5, 6]. The anaplastologist, who is commissioned to produce the tracheostomal epithesis, and the treating physician (mostly an otorhinolaryngologist) jointly perform the procedure. First, a tracheostomy tube is placed in the tracheostoma. Subsequently, the cuff, which is the inflatable balloon at the lower end of the tube, is blocked with air to avoid aspiration when the rapidly curing silicone impression material is applied by the anaplastologist in and around the tracheostomal region, including the trachea. After curing of the silicone impression material and removal of the deflated and thereby deblocked tracheostomy tube (including the cured silicone), the anaplastologist should have a perfect negative impression of the tracheostoma and a small part of the trachea (Fig. 2). Then, this cast is used for the production of a positive model of the tracheostoma, which in turn is needed for the production of the final tracheostomal epithesis. Finally, a tracheoscopy should be done to ensure that no silicone impression material has remained in the trachea or bronchi.

In the present article, we report on the case of a child who died during this procedure due to airway obstruction with silicone impression material.

## Case report

### Case history

A 13-year-old boy suffered from a severe congenital neurological disorder of unknown origin. Due to recurrent aspiration pneumonia, tracheostomy was performed, and positive-pressure ventilation via tracheostomy tube was established. Through the years, the tracheostoma-associated tissue displayed increasing irritation so that the tracheostoma became irregular and gaping. Despite multiple revision surgeries and testing of many different tracheostomy tubes, permanent ventilation without leakage could not be achieved and the cuff slipped out repeatedly during the ventilation. Therefore, the treating physicians regarded a tracheostomal epithesis as indicated to seal the gap between the tracheostomy tube and tracheostoma.

As described in the introduction, the first step in the production of a tracheostomal epithesis is the production of an impression of the tracheostoma using silicone impression material. To this end, the patient was brought to the hospital. The procedure was planned as an outpatient treatment without any anesthesia. The stomach had to be empty. The patient was lying in bed.

Reportedly, at the beginning of the procedure, the treating pediatric pulmonologist, who was regarded as experienced in this procedure, inserted a new tracheostomy tube in the tracheostoma after verification of the functionality of the cuff (cuff fills completely with air, and stays intact). The correct position of the new tracheostomy tube was checked by means of bronchoscopy. Subsequently, the tracheostomy tube was disconnected from the ventilation machine, and the cuff pressure was intensified by an additional injection of air. The patient was able to breathe for several hours without ventilatory support. The small blue control balloon outside the tracheostoma indicated that the cuff was blocked (i.e., filled with air). At this point, the vital signs of the patients were still normal.

**Fig. 3:**
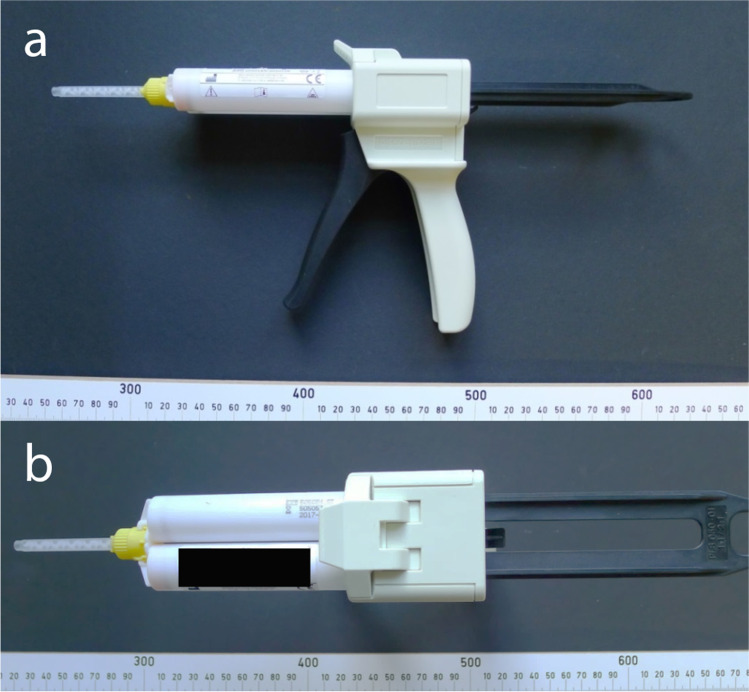
Two views (a + b) of the mixing gun with the mixing cannula (yellow). This two-component system contains two tubes with 25 ml of each component = 50 ml in one cartridge

Then, the anaplastologist pushed the tip of the mixing cannula, which was positioned on the mixing gun (Fig. 3), through the tracheostoma and syringed the pudding-like, orange-colored impression material directly next to the newly inserted tracheostomy tube. During this process, the anaplastologist wondered why the amount of the injected liquid silicone was that uncommonly high. Approximately 60 s after commencement of the procedure, pulse oximetry showed a strong decrease in oxygen saturation and, accordingly, the patient’s face turned blue.

**Fig. 4:**
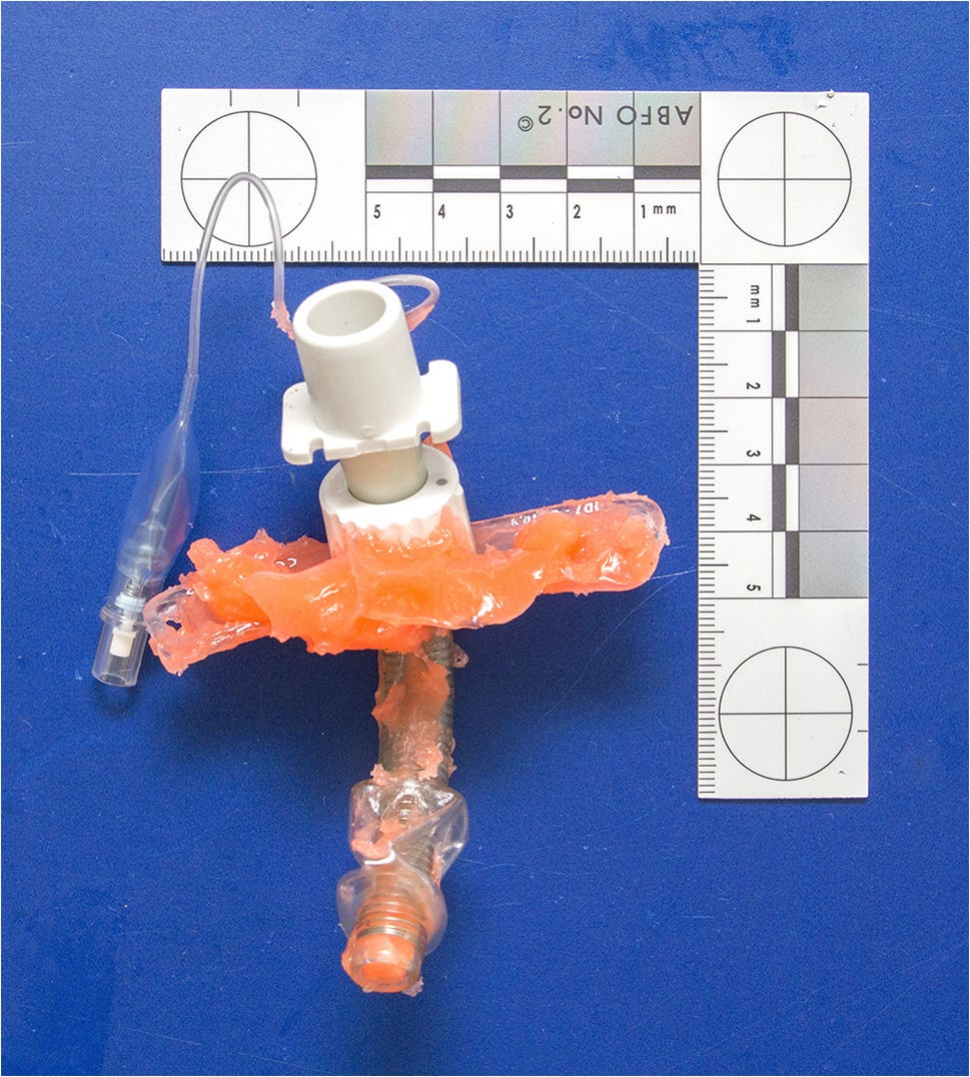
Tracheostomy tube used during the fatal incident. Note the cured orange silicone impression material which is also within the tube

**Fig. 5:**
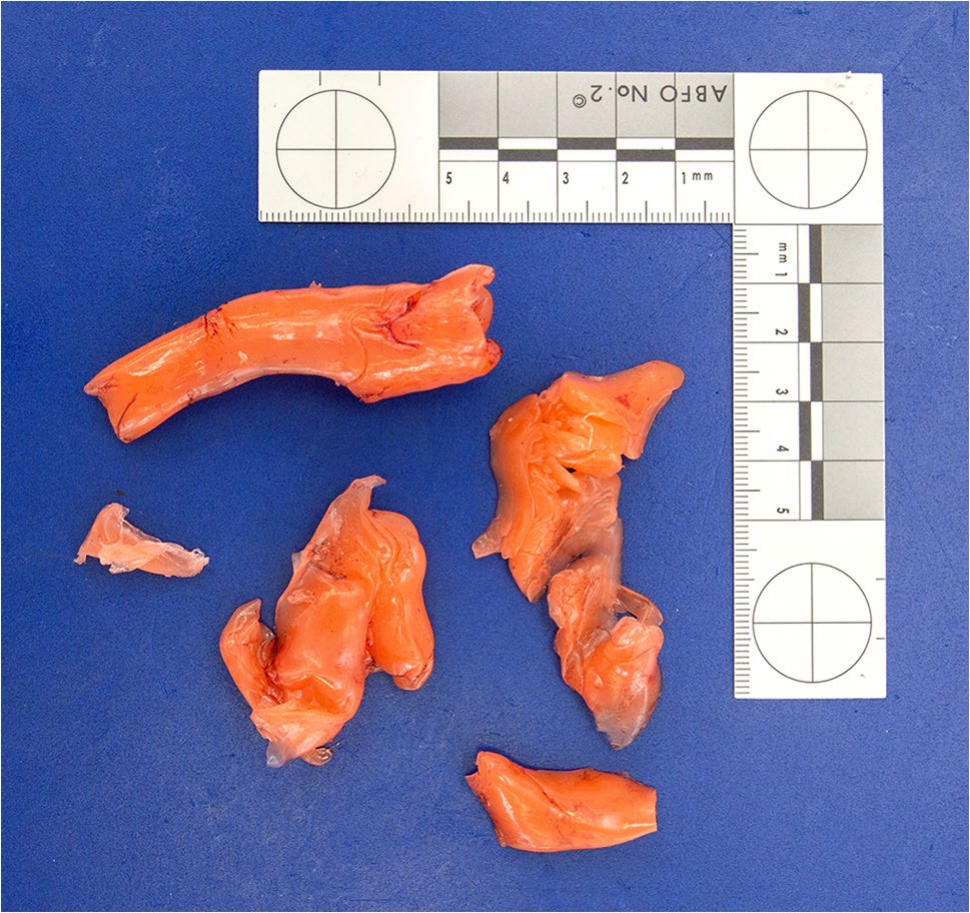
Further cured orange silicone impression material extracted from the larynx and the upper trachea

**Fig. 6:**
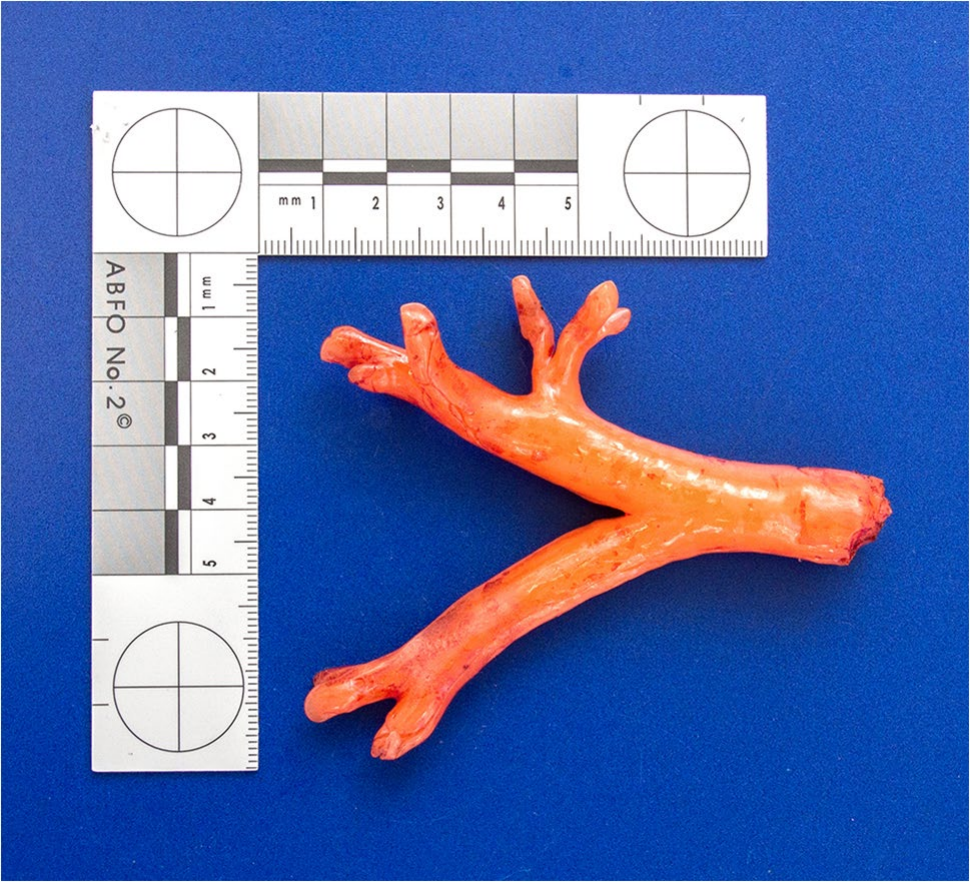
Y-shaped silicone cast extracted from the lower airways

Thereupon, the procedure was stopped immediately. The treating physicians (pediatric pulmonologist as well as an intermittently requested pediatric intensive care physician, a pediatric gastroenterologist, and an ENT specialist), started to aspirate the impression material from the trachea and administered artificial respiration using a ventilation bag. Then, the tracheostomy tube was pulled out, thereby extracting additional impression material (Fig. 4). After inserting a new tracheostomy tube, bronchoscopy corroborated additional impression material within the lower airways as well, i.e., below the former position of the cuff. Meanwhile, the patient was anesthetized and sent to the operating room. Using laryngoscopy and bronchoscopy, more moderately viscous impression material was extracted from the vocal cord area of the larynx and from the upper trachea (Fig. 5), i.e., from above the tracheostoma. Extra corporeal membrane oxygenation was established, followed by rigid bronchoscopy. After several attempts, a y-shaped silicone cast was recovered from the lower airways (Fig. 6). Despite continued resuscitation procedures, the patient died under the clinical picture of global hypoxia about 2 h after the incident had started.

### Autopsy findings

Forensic autopsy was performed 1 day after death, followed by histological and immunohistochemical investigations. At autopsy, the body of the 13-year-old boy showed a height of 140 cm and a weight of 28 kg.

According to the reported neurological disorder of unknown origin, external and internal examination revealed microcephaly, contractures of all 4 extremities, scoliosis, and a tracheostoma with an inserted tracheostomy tube.

**Fig. 7:**
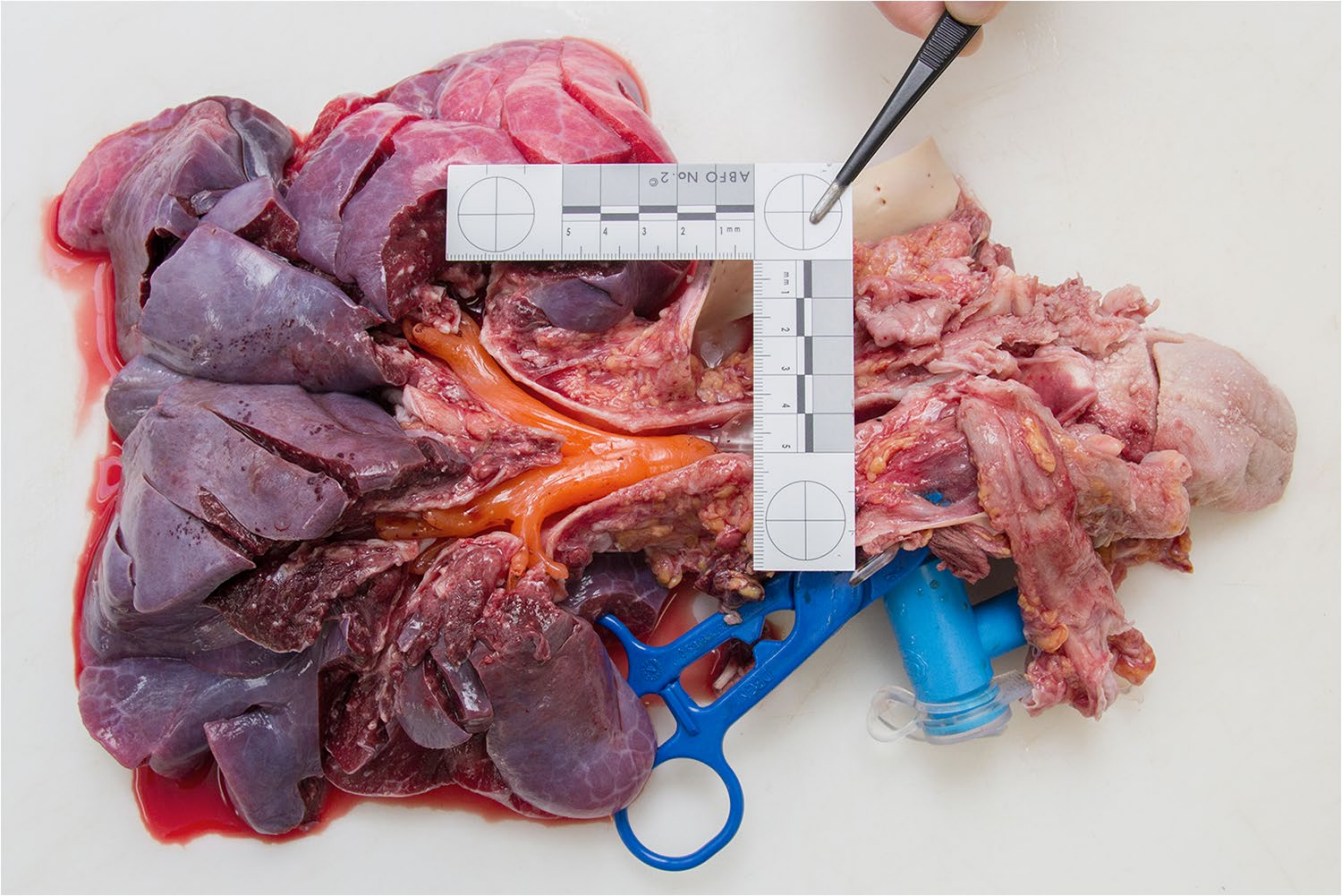
At forensic autopsy, the extracted rubberlike silicone cast of the lower airways (see Fig. 6) perfectly fitted in the lower trachea, main bronchi, and segmental bronchi of the patient

The facial skin showed no petechiae. The conjunctivae had two solitary petechiae. No residual impression material was found within the airways, neither macroscopically nor microscopically. The trachea did not show any pathological changes, especially no anatomical shape variants, widenings, or fistulae. The extracted rubberlike silicone cast of the lower airways perfectly fitted in the lower trachea, main bronchi, and segmental bronchi of the patient (Fig. 7). The lungs showed strong pulmonary edema with admixture of blood, peripheral acute emphysema as well as numerous subpleural petechiae.

The other internal organs were age-appropriately developed and did not show any pathological findings that could have promoted or caused death.

### Neuropathology

Neuropathological examination of the brain confirmed microcephaly and additionally revealed extensive calcifications, possibly due to congenital infection or a syndromic disease. Acute hypoxic damage was not yet detectable.

### Toxicology

The forensic-toxicological analysis of body liquids of the decedent proved the presence of several substances such as ketamine, atracurium, and two benzodiazepines. All qualitative and quantitative findings could be explained by the intensive care treatment during the last hours.

### Physical investigations

The impression material used in this case was a so-called addition curing impression silicone, which is regularly used in epithetics. It is a two-component system (25 ml of each component = 50 ml in one cartridge) that has to be vulcanized by using a mixing cannula and a mixing gun (Fig. 3). The volume of the cured silicone impression material extracted from the patient was 43 ml. Thus, nearly the entire content of the 50-ml cartridge was injected through the tracheostoma. At room temperature, the final formability was determined with 3–4 min, which corresponded to the manufacturer’s information. The injection required repeated manual operation of the lever of the mixing gun.

**Fig. 8:**
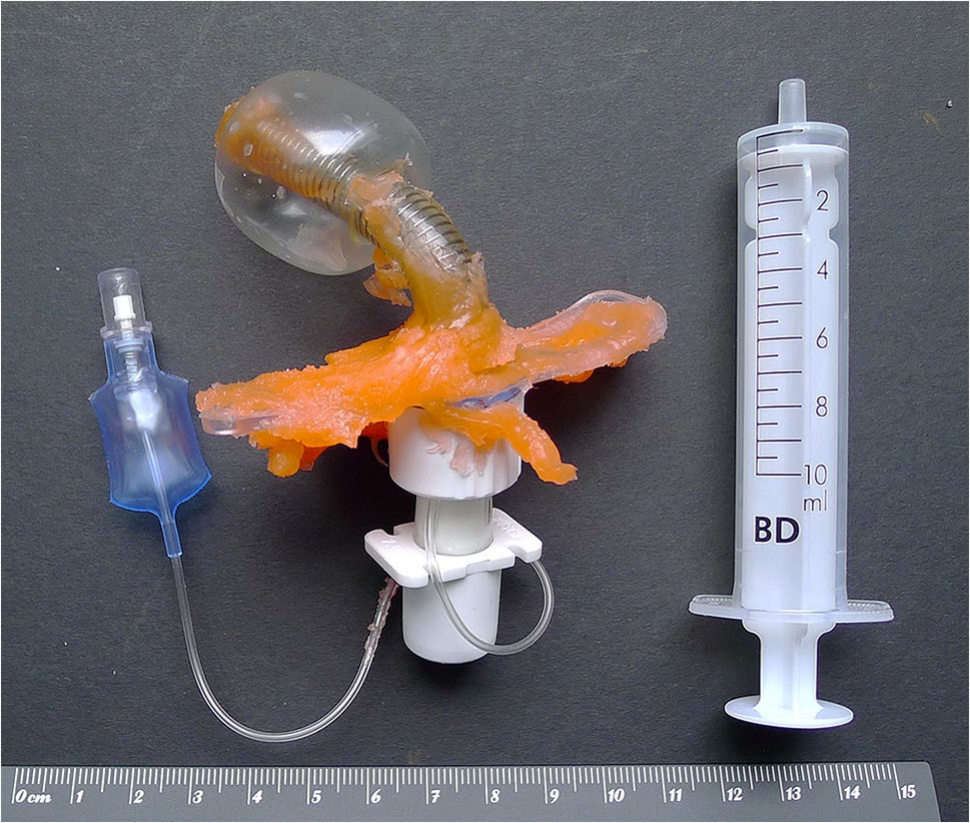
At forensic physical investigation, the cuff of the tracheostomy tube used during the fatal incident (see Fig. 4) was still able to be blocked (filled with air) properly until a maximum pressure was achieved. Note the spherical shape of this high-pressure cuff. Maximum pressure and shape was retained for several days

The cuff of the tracheostomy tube used during the injection of the silicone impression material could still be properly blocked (inflated) until a maximum pressure was achieved (Fig. 8). At maximum pressure, the cuff had a spherical shape. Maximum pressure and shape retained for several days. The small blue control balloon, which is normally outside the body, properly indicated that the cuff was blocked. This balloon may also partially serve as a compensation balloon. Just as the cuff, both the control balloon and the associated pneumatic valve, which is used for the blocking and deblocking of the cuff, did not show any functional or structural defects.

### Medico-legal conclusions

Cause of death could be determined as asphyctic suffocation, also referred to as asphyxia, due to the obstruction of the lower airways with cured silicone. Causality between the procedure described above and the cause of death had to be affirmed. There was no reasonable doubt that the viscous silicone impression material erroneously reached not only the larynx but also the lower airways beyond the cuff of the tracheostomy tube.

Apart from the tracheostoma, the trachea did not exhibit any pathological changes that could have facilitated the silicone impression material to bypass the cuff on any atypical biological way. The inserted tracheostomy tube, including the cuff at the lower end, was physically proven to be intact and fully functional. Based on these findings, it can be concluded that the cuff obviously did not prevent the passing of the injected silicone impression material. From the medico-legal point of view, there are several causes to possibly explain this conclusion:

- One possible cause could be that, contrary to statements of the persons present during the incident, the cuff was not sufficiently filled with air so that the interior space of the trachea was simply not sealed. There is no objective possibility to verify *ex post* whether the cuff was maximally blocked or not during the incident. It is only possible to confirm that, according to the forensic-physical investigations, the cuff was technically able to do this.
- Another possible cause could be that the tracheostomy tube with the intact and blocked cuff dislocated within the trachea during the procedure so that the sealing was (at least temporarily) not maintained. Accordingly, prior to the procedure, it has been reported that the cuff slipped out repeatedly during the ventilation. However, as with the first-mentioned possibility, there is no objective way to verify *ex post* whether such a situation has occurred or not. It was only possible to determine that the trachea did not show any pathological changes.
- A third possible cause could be that, although the cuff was completely blocked, the natural flexibility and elasticity of the smooth posterior wall of the trachea allowed the passage of the viscous impression material by temporary protrusions, e.g., if the pressure of the injected material was high enough to overcome the posterior wall’s natural resistance. In this case, probably the procedure itself must be questioned in principle.
- A fourth possible cause could be that the pressure of the injected material was able to overcome the resistance of the blocked cuff resulting in temporary indentations of the cuff’s wall with forming of street-like lacunae along the cuff. In this case as well, probably the procedure itself must be questioned in principle.

Moreover, any combination of the abovementioned possibilities would be conceivable as well.

### Legal assessment

The proceedings were closed by the public prosecutor’s office due to insufficient suspicion. Several reasons were given for this decision: First, the procedure was medically indicated, and informed consent was present. Second, there existed no professional guidelines for the production of a tracheostomal epithesis. Third, the anaplastologist was professionally qualified to perform the procedure because all existing trainings had been completed successfully, and more than 100 tracheostoma epitheses had been fabricated over the last 15 years. Fourth, due to intra-individual differences, it had not been possible for the anaplastologist to estimate the correct amount of silicone impression material in advance. Fifth, the medical staff had immediately recognized the emergency situation and promptly introduced the appropriate intensive-care measures. In sum, medical or other malpractice could not be determined.

## Discussion

Asphyxia in children and adolescents often involves accidents [7]. After comprehensive investigations, the present case of asphyxia due to airway obstruction with silicone impression material in a 13-year-old boy was considered an accident as well.

In the present case report, the cuff of the tracheostomy tube played a key role. The question arises of whether this small piece of plastic was suited at all for the demands required for the impression procedure, i.e., to withstand the physical characteristics of the liquid silicone impression material. In all probability, the cuff of a tracheostomy tube has not been designed and constructed for that purpose.

According to Kress [8], the cuff facilitates positive pressure ventilation and provides good protection against aspiration, meaning aspiration of material and liquids usually occurring in mechanical ventilation such as bronchial secretion or stomach contents. However, Kress also stated that no cuff is able to provide a complete protection against aspiration because the filling pressure of the cuff must not exceed certain threshold values to avoid pressure-related injuries of the tracheal mucosa [8]. Furthermore, the pressure during forceful swallowing or vomiting exceeds the permissible filling pressure of the cuff many times over so that aspiration can occur despite the cuff being correctly inserted and completely blocked [8].

The shape of the cuff represents another significant aspect described by Kress [8]. High-pressure cuffs, as required during the epithetical impression procedure, have a spherical shape. However, the contact surface of such a spherically shaped cuff to the tubularly shaped trachea is considerably lower than the cylindrical contact surface of a low-pressure cuff [8]. Hence, it is possible that the shape of the cuff additionally promoted the passage of the silicone into the lower airways.

As mentioned in the legal assessment, sufficient professional guidelines for the production of a tracheostomal epithesis did not exist at the time of the incident in 2016. The German Federal Association of Epitheticians (Deutscher Bundesverband der Epithetiker, dbve) only provides a guideline for epitheses in the head and neck area. Section 9 of the 8^th^ revised version of this guideline from 2017 deals with the impression of open wound areas such as with tracheostomas [9]. According to the dbve guideline, impressions in those wound areas are only admissible “in clinical areas” to ensure that a doctor or emergency team is able to intervene immediately if necessary. This requirement was fulfilled in the present case. Otherwise, there still exists only a registered guideline project on “craniofacial epithetics” by the Study Groups of the Scientific Medical Associations (Arbeitsgemeinschaften der Wissenschaftlichen Medizinischen Fachgesellschaften, AWMF) with a scheduled completion in June 2022 [10]. Usually, the AWMF provide professional guidelines in numerous medical fields in Germany.

To the best of our knowledge, a fatal incident during the production of a tracheostomal epithesis with obstruction of the airways with cured silicone has not been described in medico-legal literature so far. However, there are many single case reports on lethal aspirations of other more or less viscous materials. A review is presented by Koops et al. (1984) [11], describing death cases with aspiration of rare materials such as mud, cereals, fat, oil, or sawdust.

Trübner et al. (1991) [12] reported on the case of a 66-year-old and mentally disturbed woman where death by suffocation occurred due to improper usage of denture adhesive that blocked the entrance of the larynx. Similar to our case, the viscous and adhesive features of the material strongly complicated its removal and the life-saving intubation.

Morgan and Musa (2010) [13] reported on the suicide case of a 62-year-old man who died due to inhalation and ingestion of Builders Polyurethane expandable foam. The white foam occluded the upper respiratory tract down to the level of small bronchioles by forming a cast which closely resembled the cast in our case report. In addition, the mouth, pharynx, esophagus, and stomach were filled and distended by the foam as well.

Mullan and Vey (2011) [14] described the death case of a 41-year-old man who became victim of an unusual motor vehicle crash and died from drowning in another atypical medium. At autopsy, the larynx, trachea, and main bronchi appeared completely filled and obstructed by paraffin wax from the tanker trailer of the truck that the man was driving.

To conclude, the present medico-legal case report proves that the production of an impression of a tracheostoma for the purpose of the production of a tracheostomal epithesis is a procedure with a potentially lethal outcome. The application of viscous silicone impression material in and around the airways of a patient requires the absolute certainty that the obstruction of the central lower airways with cured silicone cannot occur in order to prevent death by suffocation. Therefore, from a medico-legal point of view, it should at least be ensured—e.g., by means of simultaneous tracheobronchoscopy through the inserted tracheostomy tube—that the procedure can be stopped immediately in case the impression material is yet able to pass the blocked cuff. The additional usage of a cuff pressure gauge, which was obviously not employed in the present case, should be taken into consideration to indicate and monitor the sufficient block of the cuff. Furthermore, from a forensic point of view, professional guidelines are urgently needed. These guidelines should include not only all necessary handling instructions as well as the personnel and technical prerequisites but also clear safety precautions.
